# Large‐Scale Distribution of Physical Data Using DNA‐of‐Things Technology in Newspaper Printing

**DOI:** 10.1002/smll.202511931

**Published:** 2026-03-31

**Authors:** Francesca Granito, Andreas L. Gimpel, Wendelin J. Stark, Reinhard Heckel, Robert N. Grass

**Affiliations:** ^1^ Department of Chemistry and Applied Biosciences ETH Zurich Zurich Switzerland; ^2^ Department of Computer Engineering Technical University of Munich Munich Germany

**Keywords:** DNA data storage, DNA of things, newspaper, silica nanoparticles

## Abstract

DNA represents a promising solution for high‐density and long‐term data storage. In this study, we explore the scalability and robustness of DNA‐of‐Things (DoT) technology by embedding digital data encoded in silica‐encapsulated DNA in newspaper ink. In honor of the 75th anniversary of the German Basic Law the “Grundgesetz” was encoded in DNA, encapsulated in silica nanoparticles, and mixed with paraffin‐based offset ink for mass distribution in a newspaper with a circulation of over 500 000 copies of “The Süddeutsche Zeitung.” We assessed the integrity and recoverability of the DNA after printing by retrieving, sequencing, and decoding the embedded data. Our results demonstrate the sensitivity and scalability of DNA‐of‐things technology. As a proof of concept, the DNA was reliably stored in printed media and successfully retrieved from a single dot of ink containing approximately 14 femtograms of DNA.

## Graphical abstract

DNA‐of‐things technology enables stable data storage in large‐scale newspaper printing, with successful retrieval of 37 kB of digital data from a single ink dot containing approximately 14 femtograms of DNA.

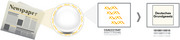

## 1 Introduction

In today's world, the question is no longer about how much data we can store, but how efficiently we can do so. As digital storage demands increase, conventional storage media encounter constraints in scalability, longevity, and security. DNA, nature's blueprint for life, emerges as a data storage alternative. While DNA for product tagging [1, 2] has so far been based on using short sequences as “barcodes,” nucleic acids are also being explored as a medium for digital data storage [3, 4, 5, 6, 7, 8]. To ensure congruency of the material's physical and digital identity, it is necessary to combine physical marking with digital tracking [9]. Various physical marking systems have been developed, including barcodes [10], laser engraving, RFID chips [11], and fluorescent markers [12, 13, 14]. While these approaches enable identification or tracking at specific stages of a product's lifecycle, they typically function as static identifiers and are often inadequate when it comes to durability, resistance to forgery, or integration across circular economies. Unlike static tags, DNA allows for encoding highly specific, unique digital identities at the molecular level [15], offering not only identification but also the potential for embedding historical data such as processing steps or thermal history [16]. DNA‐of‐Things (DoT) technology [17] bridges the gap between digital and physical data. Here, digital information is introduced as synthetic DNA into everyday objects, thereby challenging the boundaries between the digital and physical domains. This principle involves assigning the four DNA bases (A, C, T, G) to binary digits (00, 01, 10, 11), enabling the encoding of digital information inside the chemical sequence of synthetic DNA. With its theoretical storage density of several hundred exabytes per gram [3], DNA far supersedes the storage capabilities of traditional data carriers. Several research studies surrounding DNA data storage [18] have shown that stable storage over extended periods is possible through error correction [5, 7, 19] and artificial “fossilization” [5], in addition to allowing for random access in a parallel file system [6, 20]. Furthermore, molecular tools to write [21] and read [22] DNA have become increasingly available in recent years, with decreasing technological costs [23]. A key component that enables the embedding of DNA into everyday objects is the use of silica nanoparticles to encapsulate DNA strands, thereby ensuring DNA stability and protection against environmental degradation [24]. This study analyses the scalability and robustness of DNA‐of‐Things (DoT) for large‐scale information distribution in printed media. In honor of the 75th anniversary of the German Basic Law, “Grundgesetz” [25], a group of artists and activists launched the project termed “DNA of democracy” [26]. The goal was to use DNA as a conceptual bridge, connecting biological heritage with cultural legacy. The initiative aimed to raise awareness of the role of democracy and the basic human rights, which are core values within the Grundgesetz. In collaboration with the editors of the newspaper, it was decided that the 2024 Pentecost edition of the “Süddeutsche Zeitung” would include DNA encoding the text of the Grundgesetz [27].

## 2 Methods

### 2.1 General Experimental Approaches

In this report, we discuss the scientific aspects and technical challenges associated with this project. DNA‐encoded digital data was embedded within silica nanoparticles and mixed into printing ink for newspaper production. By subjecting the embedded DNA to real‐world conditions of mass printing and distribution, we assessed whether molecular data could withstand the physical and chemical stresses of large‐scale production and still be reliably retrieved. The text of the Basic Law for the Federal Republic of Germany, as presented on the website of the German federal parliament accessed in April 2024 [25], was compressed in an archive using BZip2 and encoded into DNA using the protocol presented in Meiser et al. [28]. The oligo pool was synthesized by an external service provider (Twist Biosciences). Starting with an 88‐nanogram oligo pool, the DNA was diluted and amplified using PCR with adapter sequences, as detailed in Table S1. This amplification step significantly increased the quantity of DNA, resulting in 200 micrograms of unpurified product suitable for further use. To protect the DNA and enhance its long‐term stability, the amplified oligo pool was encapsulated in silica nanoparticles using a refined method based on that of Paunescu et al. [24]. The nanoparticles maintained an encapsulation protection rate of approximately 85% for the oligo pool, as determined by PCR analysis. To enable compatibility with the hydrophobic nature of offset printing inks, the silica nanoparticles, synthesized initially in an aqueous solution, were transferred into an organic medium, allowing for uniform integration with the viscous ink formulation [29]. The resulting oil‐based suspension was then delivered to multiple printing facilities, where it was incorporated into black ink used for the front page, as well as pages 7 and 17, of over 500 000 copies of the “Süddeutsche Zeitung” [30], distributed across Germany and neighboring countries. Figure S11 shows SEM images of DNA/SiO_2_ particles in the ink suspension on paper fibers, highlighting their morphology and distribution.

**FIGURE 1:**
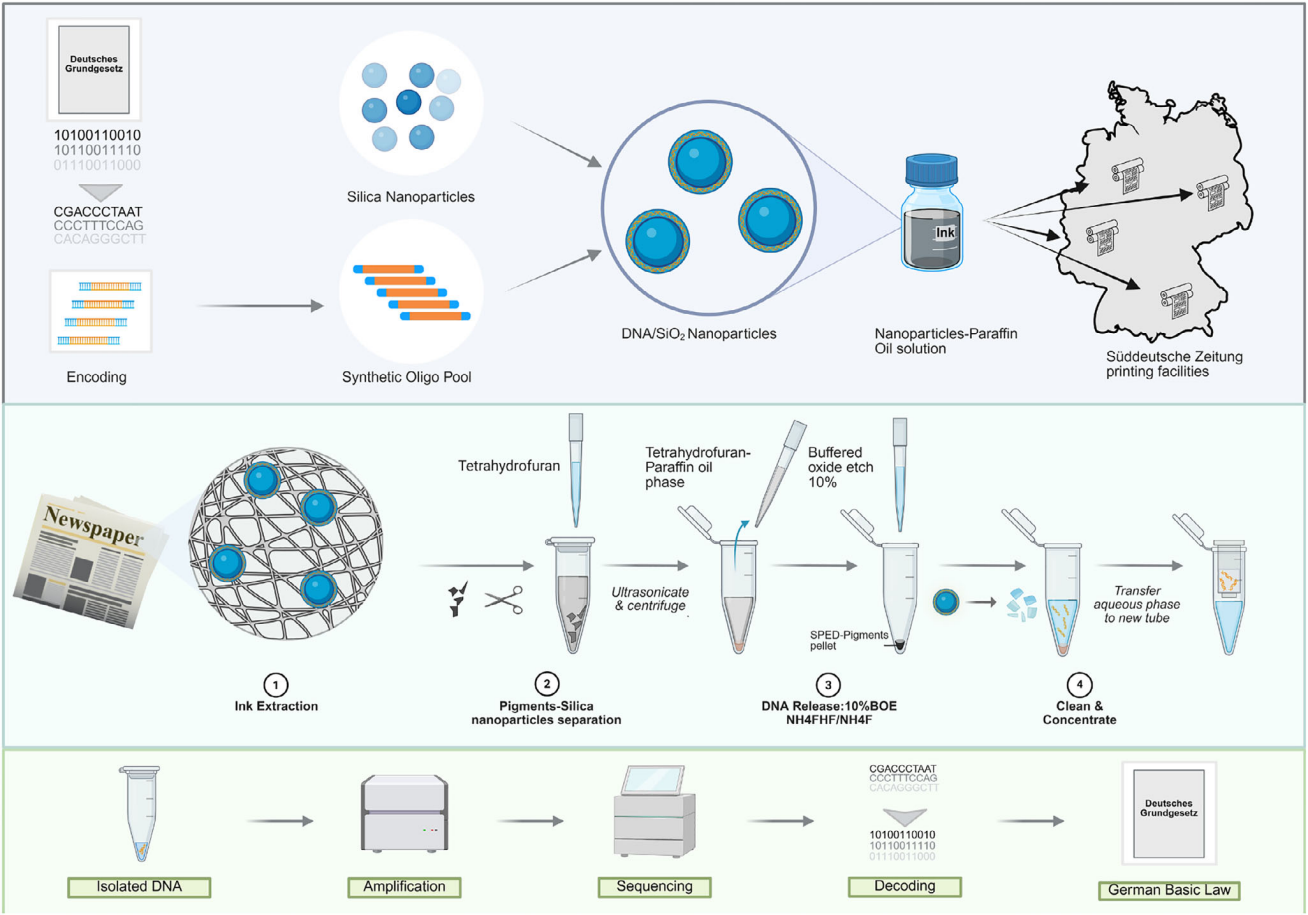
Summary of the DNA‐based information storage and retrieval mechanism. a) Encoding, synthesis, encapsulation, and distribution: Digital information is encoded into DNA sequences, synthesized into a synthetic oligonucleotide pool, and encapsulated in silica nanoparticles (SPED). The nanoparticles are then mixed into an ink formulation and used for printing, ensuring the secure distribution of DNA‐encoded data. b) Extraction and DNA retrieval: printed ink samples are subjected to ultrasonication and centrifugation to isolate the pigment‐silica nanoparticle combination. Using a buffered oxide etch (BOE) solution, DNA is liberated from the nanoparticles, then purified and concentrated. c) Extracted DNA is amplified via quantitative polymerase chain reaction (qPCR) and sequenced. Decoding of the obtained sequences allows reconstruction of the original digital information. “Figure 1” partially created in BioRender.

A central challenge was to determine the practical viability of DNA‐of‐Things technology under conditions of extreme dilution in an industrial process such as offset printing [31], over which we had no direct control, and whether the DNA concentration would be sufficient to retrieve the encoded information. To test the recoverability of the data, samples of the newspapers (NP: Zürich, Munich, and Heinsberg) were purchased from different locations and processed to enhance the ink extraction. Different sections of the print were chosen for analysis, such as picture sections, text, single words, single letters, and punctuation marks. The DNA‐containing ink was extracted using tetrahydrofuran to remove the oil‐based components in which the particles were initially suspended. The particles and pigments were then separated by centrifugation, after which the encapsulated DNA was recovered by breaking down the silica shell with a fluoride‐based etching solution, following a modified protocol by Paunescu et al. [24]. A subsequent centrifugation step isolated the supernatant fraction containing the DNA oligo pool. This method enabled the controlled release of DNA without compromising its molecular integrity, allowing for accurate downstream analysis. The extraction protocol was further optimised, the DNA recovery rate was improved by around two orders of magnitude (118‐fold, see Figure S3). Despite these improvements, pigments and paper residues in the extracted solution continued to interfere with qPCR analysis. To overcome this, an additional purification and concentration step was introduced at the end of the extraction process, effectively removing inhibitors and enhancing DNA recovery (see Note S1). Figure 1 provides an overview of the entire process, from encoding and extraction to retrieval and decoding of the information.

### 2.2 Encoding and Oligonucleotide Pool Preparation

The text of the Basic Law for the Federal Republic of Germany, as presented on the website of the German federal parliament [25], was stored in an archive using BZip2 compression and encoded into DNA using the protocol presented in Meiser et al. [28]. Specifically, the archive was encoded using the parameters “–n = 2000 –k = 1755 –N = 36”, resulting in 2000 sequences of 108 nucleotides each with an outer redundancy of 13.9% and inner redundancy of 12.5%. These sequences were ordered from Twist Bioscience as an oligonucleotide pool. After resuspension to 10 ng µL^−1^ and dilution by 5000x using ultrapure water, the pool was amplified for 24 cycles in a 96‐well plate using PCR (see below). The amplified DNA was used without additional purification.

### 2.3 Encapsulation of DNA In Silica Nanoparticles

The amplified DNA was encapsulated in commercial silica nanoparticles similarly to the protocol presented in Paunescu et al. [24]. Specifically, 1 mL of silica nanoparticles—SiO_2_‐R‐0.15 (143 nm, 50 mg mL^−1^ microParticles GmbH) were pelletized and suspended in 1 mL isopropanol (99.9%, Sigma–Aldrich). After ultrasonication, 100 µL N‐trimethoxylsilylpropyl‐N,N,N‐trimethylammonium chloride (TMAPS, 50% w/v in methanol, ABCR) were added, and the suspension shaken at 900 rpm for 12 h in a thermoshaker. After washing and resuspending in 1 mL isopropanol, 70 µL of the functionalized silica nanoparticles were added to 1 mL of amplified DNA (100 ng µL^−1^) together with 20 µL of polyvinylpyrrolidone solution (10 g L^−1^). After a few minutes, the particles were pelletized and resuspended in 0.5 mL of ultrapure water. To this suspension, 0.5 µL TMAPS and 0.5 µL tetraethoxysilane (TEOS, ≥99.0%, Sigma–Aldrich) were added, and the suspension was shaken at 900 rpm. After 4 h, an additional 4 µL TEOS were added. Shaking was stopped after four days, and the particles were pelletized and finally resuspended in 0.5 mL ultrapure water. For the newspaper ink, a total of four batches were combined to yield 2 mL of silica‐encapsulated DNA.

### 2.4 Newspaper Printing

To formulate the silica‐encapsulated DNA for cold‐set printing, the particles were pelletized and resuspended in 1 mL tetrahydrofuran (99.9%, Sigma–Aldrich). 150 µL of this suspension were then added to 20 mL paraffin oil (Merck Millipore), which was then mixed for 1 min at 3000 rpm in a speed mixer (MX‐F Vortex Mixer, 2500 rpm). The suspension was then transferred to 50 mL Schott flasks for shipping. At each printing facility the suspension was mixed into 34 kg of commercial black offset ink (Hubergroup 39GN106 GOOD NEWS Schwarz). This process was performed for each of the four printing locations used for printing pages 1, 7, and 17. The employed printing technology was commercial cold‐set web offset printing. In this technology, which is typical for newspaper printing, the ink is transferred to the paper via a printing plate and the ink dries through adsorption into the paper.

### 2.5 DNA‐of‐Things Ink and Inkjet Printing

For inkjet printing experiments, silica‐encapsulated DNA particles were introduced into Epson 104 Ink Series Genuine Eco Tank (SKU:C13T00P140) black ink. A 50 µL aliquot of a 7 g L^−^^1^ nanoparticle stock solution was centrifuged and resuspended in 50 µL of 99% ethanol (Sigma‐Aldrich). The particles were then mixed with 15 mL of ink, followed by gentle mixing to ensure homogeneity. Printing was performed using an Epson ET‐2860 inkjet printer under standard operating conditions using conventional inkjet printing paper. No nozzle clogging, print defects, or operational instability were observed during printing.

### 2.6 Recovery of Silica‐Encapsulated DNA From Newspaper

For the recovery of silica‐encapsulated DNA, different sections of different sizes were sampled. Sections weighing 40 mg each were taken from figures, text, a word, a single letter, and a dot. A negative control sample from a different newspaper (“20 Minuten”) was included to monitor cross‐contamination. All samples were stored and handled together. The newspaper samples were shredded to increase the available surface area and washed in 1 mL of tetrahydrofuran (THF). After ultrasonication, the THF solution containing extracted ink was separated from the paper and centrifuged at 21 500 × g for 4 min at room temperature. The resulting pellet, composed of ink pigments and silica particles, was washed once with 99% ethanol (Sigma–Aldrich). Silica dissolution was carried out using fluoride‐containing solutions following the protocol described by Paunescu et al. [24]. A mixture of 0.23 g NH_4_HF (>98%, reinst, Carl Roth GmbH) and 0.19 g ammonium fluoride (>98%, ACS Reagent, Sigma Aldrich Co) was prepared with 10 mL of Milli‐Q ultrapure water (type 1, 18.2 MΩ·cm at 25°C) to create a buffered oxide etch. A volume of 10 µL of buffered oxide etch was added to the particle pellet, followed by vortexing and the addition of 100 µL Milli‐Q water. The concentrations and pH of the etchants were chosen to prevent hydrolysis or denaturation of nucleic acids during the release step. The solution was vortexed again and subjected to ultrasonication for 10 min at 25°C, dissolving the protective coating and fully degrading the silica particles. The resulting black‐colored solution was centrifuged at 21 500 × g for 4 min at room temperature. In this step, DNA remained in the supernatant, while pigments formed the pellet. The supernatant was collected, purified using the DNA Clean & Concentrator PCR purification kit (Zymo Research, cat. no. D4004), and concentrated to a final volume of 15 µL.

### 2.7 Recovery of Silica‐Encapsulated DNA from Inkjet‐Printed Paper

For downstream DNA recovery and analysis, printed paper samples (50 mg) were collected. To monitor potential cross‐contamination, samples of white conventional inkjet printing paper were included; all samples were stored and handled together. The printed paper samples were shredded to increase the available surface area and washed in 1 mL of tetrahydrofuran (THF). After ultrasonication, the THF solution containing the extracted ink was separated from the paper, transferred to a separate 1.5 mL tube, and centrifuged at 21 500 × g for 4 min at room temperature. Subsequently, 1 mL of Milli‐Q ultrapure water (type 1, 18.2 MΩ·cm at 25°C) was added to the previously washed paper. Following ultrasonication, the aqueous solution containing the remaining extracted ink was separated from the paper and added to the pellet obtained from the THF extraction. The combined sample was ultrasonicated, vortexed, and centrifuged at 21 500 × g for 4 min at room temperature. A volume of 10 µL of buffered oxide etch was added to the resulting pellet, followed by vortexing and the addition of 100 µL Milli‐Q water. The solution was vortexed again and subjected to ultrasonication for 10 min at 25°C, resulting in dissolution and degradation of the silica particles. The resulting solution was centrifuged at 21 500 × g for 4 min at room temperature. The supernatant was collected, purified using the DNA Clean & Concentrator PCR purification kit (Zymo Research, cat. no. D4004), and concentrated to a final volume of 15 µL.

### 2.8 Quantification by PCR

For PCR, 5 µL of a sample, and 1 µL each of the forward and reverse primers (0 F/0 R, 10 µm), Table S1, were added to 10 µL of 2x KAPA SYBR FAST polymerase master mix (Sigma–Aldrich), unless noted otherwise. Ultrapure water was always added up to a final volume of 20 µL. For thermal cycling, an initial denaturation 5 min, 95°C, followed by 15 s, 54°C for 30 s, and 72°C for 30 s was used, for 40 cycles. Primers were purchased from Microsynth and diluted as required with ultrapure water.

### 2.9 Recovery of Silica‐Encapsulated DNA

Sequencing preparation was performed using the protocol presented in Meiser et al. [28]. Specifically, after the quantitative PCR, duplicates of the same sample were pooled and purified using spin column purification (DNA Clean & Concentrator‐5, Zymo Research). After elution with ultrapure water, samples were first amplified using 1F/1R primers (Table S1) for 12 cycles, and then purified by excising the appropriate band on an E‐Gel EX agarose gel (2%, Invitrogen), and purification by spin column (Zymoclean Gel DNA Recovery Kit, Zymo Research). This process was repeated to introduce sequencing indexes using 2F/2RI primers (Tables S2 and S3). After purification, all samples were individually diluted to 1 nm, pooled, and sequenced on an Illumina iSeq 100 sequencer according to the manufacturer's instructions.

### 2.10 Statistical Analysis

All extraction experiments were performed in two experimental duplicates, and each duplicate was analyzed in two technical replicates (e.g., qPCR measurements). Data are presented as mean ± standard deviation (SD) calculated across these replicates. No transformation or normalization was applied. Errors reflect the variability among the combined experimental and technical replicates. Raw data for all measurements are provided in the Supporting Information. Descriptive statistics (mean ± SD) are provided to illustrate reproducibility and variability of the measurements.

### 2.11 Decoding of the Data from DNA

The demultiplexed sequencing data was post‐processed by read merging using NGmerge [32] (v0.3) using the options “‐m 10 ‐d ‐e 10.” Decoding of the data was performed as described by Meiser et al. [28], using the aforementioned options for the codec. The analysis of errors was performed using DT4DDS (v1.1) by Gimpel et al. [33].

## 3 Results

**FIGURE 2:**
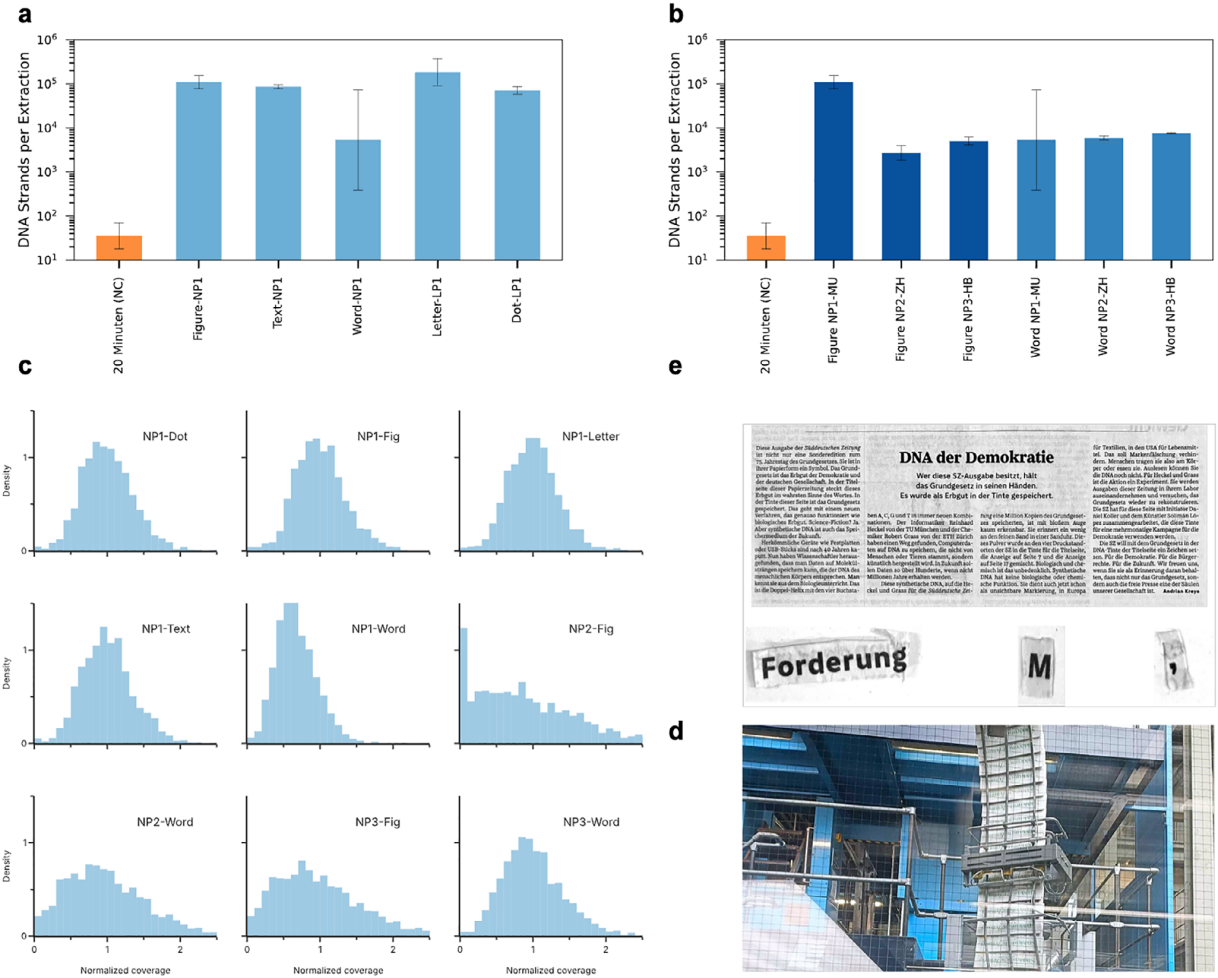
a) Number of DNA strands retrieved per extraction, respectively. A Swiss newspaper “20 Minuten” was used as a negative control, followed by different portions of the newspaper from a piece of a figure to a single dot, (raw data and calibration curve available in Figures S4 and S5). b) Number of DNA strands retrieved per extraction present in different portions of two newspapers purchased in distinct geographical areas, (raw data and calibration curve available in the Figures S4 and S5). c) Normalized coverage distributions. A wider distribution reflects unequal representation of oligos, increasing the likelihood of losing low‐coverage sequences and highlighting variability in the DNA retrieval process. d) Photographs of sections of Newspaper. e) Photograph of the primary printing facility for the Süddeutsche Zeitung, located in Munich‐Steinhausen. NP1–3 correspond to newspapers purchased in Munich, Zurich, and Heinsberg, respectively. Data are presented as mean ± SD from two experimental duplicates, each with two technical replicates; details on sample size and statistical analysis are provided in the Statistical Analysis subsection. Image of Newspaper Süddeutsche Zeitung GmbH, Munich. Reprinted with kind permission of Süddeutsche Zeitung Content.

To evaluate the reliability of retrieving information from printed DNA‐encoded materials, we first tackled the challenge of identifying the minimum physical sample size needed for successful DNA recovery. After extraction, the DNA was amplified using quantitative PCR to measure concentration, extraction efficiency, and degradation levels. This enabled us to systematically test whether the DNA oligo pool could be retrieved from increasingly smaller printed samples. The outsourcing of the embedding process introduced variability, offering an opportunity to assess the system's robustness under real‐world production and distribution conditions. Remarkably, successful DNA recovery was achieved even from single ink dots on newspaper samples, as shown in Figure 2a, demonstrating the method's high sensitivity. In all experiments, a different newspaper, “20 Minuten,” was used as a negative control; the newspapers were stored and handled together to monitor for cross‐contamination.

Interestingly, DNA concentrations remained consistent across samples of varying size, which was not expected. Further investigation revealed that the presence of excess printing paper inhibited PCR amplification, limiting the reaction efficiency and masking any concentration differences between samples. This inhibitory effect is further discussed in the Figures S1 and S2 and Note S1. To evaluate the potential for large‐scale deployment, we tested DNA recovery from various editions of the Süddeutsche Zeitung, which were printed and distributed across different locations (e.g., Zürich, Munich, and Heinsberg). Figure 2b provides evidence of the reliable recovery of the encoded data, confirming the reproducibility of both the embedding and extraction processes under real‐world printing conditions.

**FIGURE 3:**
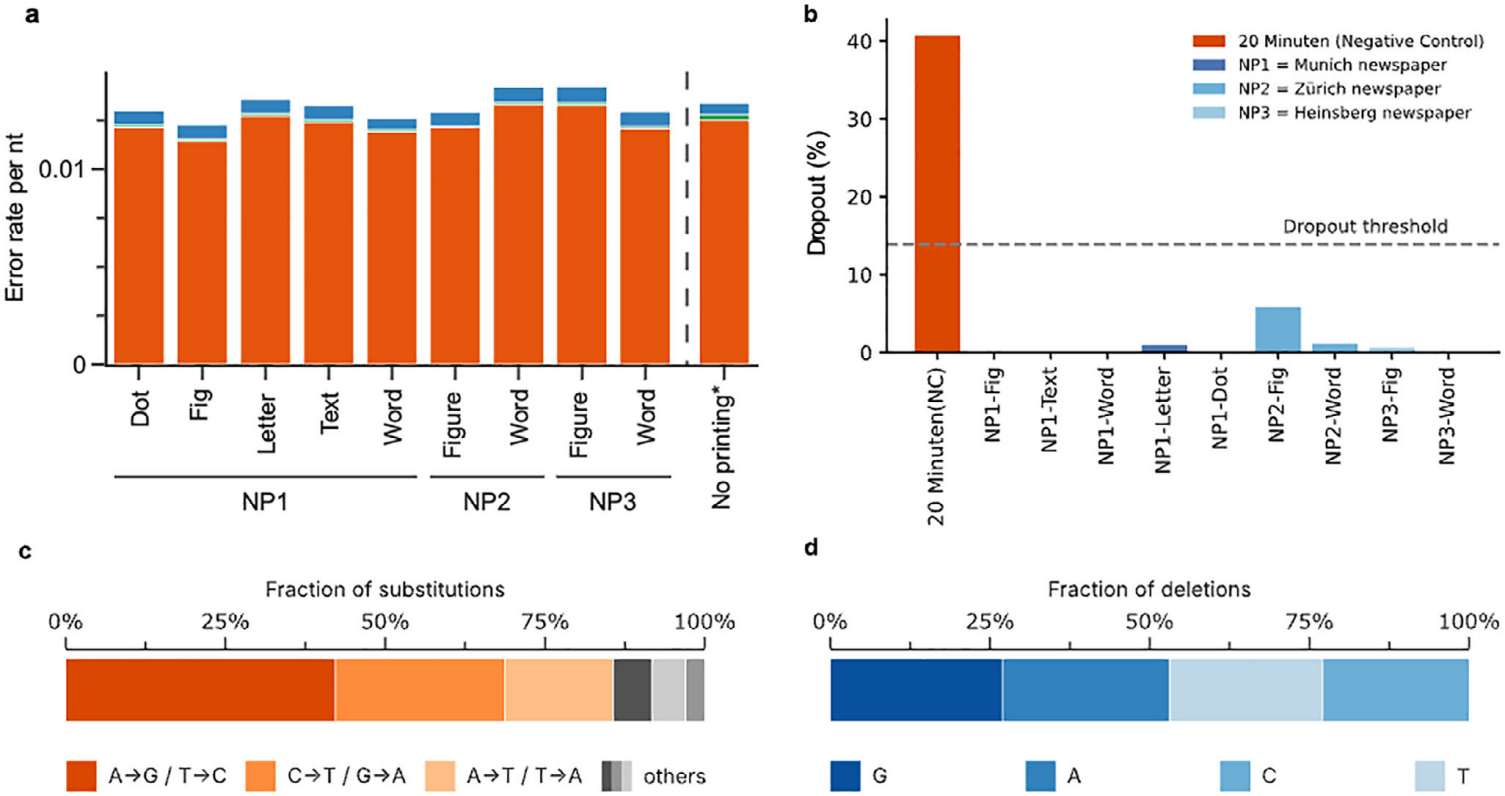
Errors (a) Overall error rate for each experiment (orange: substitutions, blue: deletions, green: insertions), *compared to the DNA data storage error rate without printing [33]. (b) Error rate by position. (c) Substitution errors. (d) Deletion errors.

To retrieve the encoded information, we performed DNA sequencing using Illumina iSeq technology. Following the extraction procedure, we obtained, for a single dot, a theoretical yield of 14 femtograms of DNA, corresponding to approximately 30 copies of the encoded file. PCR analysis measured 11 femtograms, indicating an extraction efficiency of about 80%. Detailed calculations are provided in Tables S4 and S5, while Table S6 compares the theoretical yield with the measured DNA concentration for a single dot of ink. Sequencing the library with the iSeq produced an average of 356 117 successfully merged reads per experiment. The average sequencing depth was approximately 180, calculated by dividing the average number of merged reads by the number of sequences. The demultiplexed sequencing data were post‐processed by read merging using NGmerge [32] (v0.3). Decoding of the data was performed as described by Meiser et al. [28], using the specific codec. The analysis of errors was performed using DT4DDS (v1.1) and based on the framework proposed by Gimpel et al. [33]. Error analysis was conducted to evaluate dropout rates, coverage distribution, and overall error rates, as shown in Figure 3.

We successfully retrieved the encoded file from all tested samples, various sizes and types of newspapers. To explore how mass distribution affects library composition, we analyzed coverage distribution and sequencing error rates. Figure 2c shows the normalized coverage of the DNA oligo pool across all experiments. A wider distribution indicates unequal representation of oligos, which increases the chance of losing low‐coverage sequences. Across all sequencing rounds, the overall sequence dropout rate (fraction of sequences not sequenced after extraction) remained below 6% for all samples, as shown in Figure 3b. No correlation was observed between the surface area of the extracted newspaper and the dropout level. This can be attributed to the presence of paper additives that interfere with PCR amplification, as illustrated in Figure S2. The negative control sample, “20 Minuten,” exhibited a dropout rate exceeding 40%, which is significantly higher than the “Dropout threshold” of 13.9%, and indicates that processing the negative control together with the actual samples resulted in some minor contamination, the DNA found in the negative control was not sufficient to decode the file. The dropout threshold corresponds to the maximum redundancy provided by the error correction code, above which it is no longer possible to decode the stored information reliably. We also measured sequencing error rates, including substitutions, insertions, and deletions. Overall, per‐nucleotide error rates were low (Figure 3a), remaining below 0.015, which is comparable to those reported by Gimpel et al. [33]. (No printing*, Figure 3a), for Twist oligo synthesis and Illumina sequencing, indicating that the printing process does not introduce a measurable increase in sequence errors beyond those inherent to established DNA synthesis and sequencing workflows. Among these, substitution errors were the most frequent, which can be attributed to the use of a Taq‐based polymerase known to introduce substitutions at a rate of approximately 1.09 × 10^−^^4^ nt^−^^1^ per cycle [33]. This polymerase exhibits a strong bias toward A→G and T→C transitions (61% of substitutions), with a secondary enrichment for A→T and T→A transversions (13%), consistent with previously reported error patterns, as described by Gimpel et al. [33, 34, 35]. To further reduce errors, merged reads were clustered to generate consensus sequences, minimizing residual sequencing errors of one order of magnitude [36, 37], as described in Note S2 and Figure S7.

Additionally, we assessed the position‐specific error rates averaged over all experiments (see Figure S6). In all experiments, higher error rates (up to 0.15 errors per nt) are observable for the index region. This section of the sequences, which encode for a running number result in a low‐diversity area of the DNA pools, which pose a higher challenge to the sequencer [38] and thereby higher error rates. However, these substitution errors can be handled well by the inner decoder, which at the chosen redundancy has a theoretical capacity of correcting two substitution errors per sequence. Substitution error ratios were classified by type. Due to the limitations of PCR, complementary substitution patterns cannot be distinguished (e.g., an A→G substitution may reflect a T→C substitution on the complementary strand). Deletion errors were also analyzed by base type. They were found to occur with roughly equal frequency across all nucleotides, suggesting a likely link to the synthesis process [39]. The overall low error rates, shown in Figure 3, combined with the observed substitution‐dominant pattern, reflect the expected interplay between polymerase fidelity, PCR‐induced biases, and sequencing artefacts [19, 33, 40].

To show that the DNA‐of‐things technology is not only compatible with newspaper printing, but also with other printing technologies, we also performed tests using an inkjet printer, and performed UV and thermal aging‐tests of the printed DNA. For the ink‐jet printer, an ethanolic suspension of DNA‐loaded silica nanoparticles was added to the black ink of a commercial ink‐jet printer (Epson ET‐2860 inkjet printer), and the printer was utilized without any further modification to print text on standard A4 paper. No clogging of the printing device was observed during the experiments, which included the operation of the ink‐jet printer over several days and the printing of 150 pages. The printed paper was subjected to very harsh stability tests, including abrasion tests using P120 sandpaper, UVC irradiation, and accelerated aging at 70°C over the course of six days, which is equivalent to roughly 164 years at 20°C [5] (Note S3). In all cases, the amount of DNA extractable after the aging test was only slightly distinguishable from the original concentrations (Figures S8–S10), displaying the robustness of storing digital data as DNA in printed paper.

In terms of practical applicability, DNA‐of‐things technologies are still hampered by costs. It has previously been shown that the technology is compatible with > 1 MB data quantities and various digital data formats, including audio and video [41] but the cost of DNA synthesis remains considerable. At a pricing of array‐based DNA synthesis of about 0.0001 USD per nucleotide [42] this relates to ca. 500 USD / MB of data [17, 43] assuming an encoding density of 1.5 bit/nt. This remains several orders of magnitude more expensive than conventional storage media, where tape storage only costs 16 USD per terabyte [44] (=0.000016 USD/MB). In contrast to conventional media, DNA data storage incurs a one‐time synthesis cost, while copying via PCR is essentially free, costing less than USD 1 per well and enabling the generation of billions of copies. This cost structure, high upfront cost and low replication cost, closely mirrors offset newspaper printing, which relies on expensive plate preparation but minimal per‐copy cost. Unlike QR codes, DNA‐of‐things requires no additional printing space, an important advantage in mass media where space is costly, as reflected in the high price of print advertisement. While QR codes currently offer a practical solution for small data payloads (up to ca. 3kB), advances in ubiquitous sequencing could expand the applicability of DNA‐based information storage in the near future [45].

## 4 Conclusion

The potential of DNA‐of‐Things (DoT) technology has been previously demonstrated by Koch et al. [17], who successfully embedded digital information into physical objects without compromising their functionality. The applications of this technology range from molecular steganography to more speculative uses, such as self‐replicating systems. As summarized in Table 1, previous studies have demonstrated the storage of diverse types of information, ranging from short text messages to images, 3D models, and complete video files, using different encoding strategies, storage media, and compression methods. These approaches span a wide range of file sizes, data densities, and replication scales, from a small number of experimental copies to hundreds of thousands of mass‐distributed instances.

**TABLE 1:** Comparison of representative DNA data storage studies. The table contrasts the type of information encoded, the size of the stored files, and the very different scales at which data was replicated or distributed.

Study/System	What was stored	File size	Copies/Instances
**Clelland et al., *Nature* (1999)** “Hiding messages in DNA microdots” [46]	Secret message: “June 6 invasion: Normandy”	—	1, 10, 100 copies tested; recovered after postal mailing on paper
**Shipman et al., *Nature* (2017)** “CRISPR–Cas encoding of a digital movie into the genomes of a population of living bacteria” [47]	Image frames: Human hand GIF: Muybridge horse animation.	784 bytes 3.6 KB (5 frames)	Data replicated 48 bacterial generations
**Organick et al., *Nat. Biotech* (2018)** “Random access in large‐scale DNA storage” [20]	35 digital files (text, images, video)	200+ MB (files: 29 KB–44 MB)	Entire pool synthesized; random file access via primers
**Koch et al., *Nat. Biotech* (2019)** “DNA‐of‐Things: Embedding DNA memory in objects” [17]	Stanford Bunny (3D model) + Video clip	45 KB + 1.4 MB	DNA embedded in silica beads within 3D‐printed objects; recovered over 5 print generations
**This work (2025)** *DNA of Democracy*	Basic Law (Grundgesetz)	36,847 bytes	DNA embedded in 500 000 copies of the Süddeutsche Zeitung newspaper

This work represents the largest implementation of DoT to date, embedding the full text of a legal document into 500,000 printed newspapers. We demonstrate that DNA can be extracted reliably from ink at concentrations of 14.4 picograms of DNA per gram of newspaper retrieved from a single ink dot (around 14 femtograms of DNA). These results emphasize the feasibility of DoT for mass deployment and highlight new opportunities in secure printing, anti‐counterfeiting, and distributed digital archiving. Continuous improvements in DNA synthesis [48], sequencing, and error correction [5, 7, 19, 28] will further facilitate the integration of DNA‐based storage into both scientific and commercial sectors [20]. As DNA data storage technologies advance, we can expect to see new applications emerge in areas such as digital security, product traceability, and sustainable information management.

## Funding

Funded by ETH Zurich and the European Union (DiDAX 101115134). Views and opinions expressed are however those of the author(s) only and do not necessarily reflect those of the European Union or the European Research Council Executive Agency. Neither the European Union nor the granting authority can be held responsible for them. Swiss Participants in this project are supported by the Swiss State Secretariat for Education, Research and Innovation (SERI) under contract number 23.00330.

## Conflicts of Interest

RNG and WJS are inventors of a patent concerning the encapsulation of DNA in silica nanoparticles. This patent is licensed to Haelixa AG, and RNG and WJS are minority Haelixa shareholders.

## Supporting information




**Supporting File**: smll73270‐sup‐0001‐SuppMat.docx.

## Data Availability

The data that support the findings of this study are openly available in Figshare at https://doi.org/10.6084/m9.figshare.c.8017144, reference number 8017144.

## References

[1] A. Galimberti , F. De Mattia , A. Losa , et al., “DNA Barcoding as a New Tool for Food Traceability,” Food Research International 50, no. 1 (2013): 55–63, 10.1016/j.foodres.2012.09.036.

[2] W. J. Kress and D. L. Erickson , “Methods in Molecular Biology,” DNA Barcodes: Methods and Protocols, (Humana Press, 2012), 858, 10.1007/978-1-61779-591-6. 22684949

[3] G. M. Church , Y. Gao , and S. Kosuri , “Next‐Generation Digital Information Storage in DNA,” Science 337, no. 6102 (2012): 1628–1628, 10.1126/science.1226355. 22903519

[4] N. Goldman , P. Bertone , S. Chen , et al., “Towards Practical, High‐Capacity, Low‐Maintenance Information Storage in Synthesized DNA,” Nature 494, no. 7435 (2013): 77–80, 10.1038/nature11875. 23354052 PMC3672958

[5] R. N. Grass , R. Heckel , M. Puddu , D. Paunescu , and W. J. Stark , “Robust Chemical Preservation of Digital Information on DNA in Silica With Error‐Correcting Codes,” Angewandte Chemie International Edition 54, no. 8 (2015): 2552–2555, 10.1002/anie.201411378. 25650567

[6] S. M. Hossein , T. Yazdi , Y. Yuan , J. Ma , H. Zhao , and O. A. R. Milenkovic , “A Rewritable, Random‐Access DNA‐Based Storage System,” Scientific Reports 5, no. 1 (2015): 14138, 10.1038/srep14138. 26382652 PMC4585656

[7] Y. Erlich and D. Zielinski , “DNA Fountain Enables a Robust and Efficient Storage Architecture,” Science 355, no. 6328 (2017): 950–954, 10.1126/science.aaj2038. 28254941

[8] K. J. Tomek , K. Volkel , A. Simpson , et al., “Driving the Scalability of DNA‐Based Information Storage Systems,” ACS Synthetic Biology 8, no. 6 (2019): 1241–1248, 10.1021/acssynbio.9b00100. 31117362

[9] T. Rumetshofer and J. Fischer , “Information‐Based Plastic Material Tracking for Circular Economy—A Review,” Polymers 15, no. 7 (2023): 1623, 10.3390/polym15071623. 37050237 PMC10097355

[10] GS1 Barcodes, accessed August 09, 2024, https://www.gs1.org/standards/barcodes.

[11] B. Unhelkar , S. Joshi , M. Sharma , S. Prakash , A. K. Mani , and M. Prasad , “Enhancing Supply Chain Performance Using RFID Technology and Decision Support Systems in the Industry 4.0–A Systematic Literature Review,” International Journal of Information Management Data Insights 2, no. 2 (2022): 100084, 10.1016/j.jjimei.2022.100084.

[12] J. Woidasky , I. Sander , A. Schau , et al., “Inorganic Fluorescent Marker Materials for Identification of Post‐Consumer Plastic Packaging,” Resources, Conservation and Recycling 161 (2020): 104976, 10.1016/j.resconrec.2020.104976.

[13] I. Jakubowicz and N. Yarahmadi , “Review and Assessment of Existing and Future Techniques for Traceability With Particular Focus on Applicability to ABS Plastics,” Polymers 16, no. 10 (2024): 1343, 10.3390/polym16101343. 38794535 PMC11124994

[14] X.‐S. Li , W.‐P. He , L. Lei , et al., “Laser Direct Marking Applied to Rasterizing Miniature Data Matrix Code on Aluminum Alloy,” Optics & Laser Technology 77 (2016): 31–39, 10.1016/j.optlastec.2015.08.020.

[15] A. X. Kohll , J. Koch , W. D. Chen , et al., “DNA Barcode Quantification as a Robust Tool for Measuring Mixing Ratios in Two‐Component Systems,” ACS Applied Bio Materials 2, no. 11 (2019): 5062–5068, 10.1021/acsabm.9b00735. 35021504

[16] D. Paunescu , R. Fuhrer , and R. N. Grass , “Protection and Deprotection of DNA—High‐Temperature Stability of Nucleic Acid Barcodes for Polymer Labeling,” Angewandte Chemie International Edition 52, no. 15 (2013): 4269–4272, 10.1002/anie.201208135. 23468228

[17] J. Koch , S. Gantenbein , K. Masania , W. J. Stark , Y. Erlich , and R. N. Grass , “A DNA‐of‐Things Storage Architecture to Create Materials With Embedded Memory,” Nature Biotechnology 38, no. 1 (2020): 39–43, 10.1038/s41587-019-0356-z. 31819259

[18] L. Organick , B. H. Nguyen , R. McAmis , et al., “An Empirical Comparison of Preservation Methods for Synthetic DNA Data Storage,” Small Methods 5, no. 5 (2021): 2001094, 10.1002/smtd.202001094. 34928102

[19] R. Heckel , G. Mikutis , and R. N. Grass , “A Characterization of the DNA Data Storage Channel,” Scientific Reports 9, no. 1 (2019): 9663, 10.1038/s41598-019-45832-6. 31273225 PMC6609604

[20] L. Organick , S. D. Ang , Y.‐J. Chen , et al., “Random Access in Large‐Scale DNA Data Storage,” Nature Biotechnology 36, no. 3 (2018): 242–248, 10.1038/nbt.4079. 29457795

[21] S. Kosuri and G. M. Church , “Large‐Scale De Novo DNA Synthesis: Technologies and Applications,” Nature Methods 11, no. 5 (2014): 499–507, 10.1038/nmeth.2918. 24781323 PMC7098426

[22] National Human Genome Research Institute , “DNA Sequencing Costs: Data,” accessed August 21, 2024, https://www.genome.gov/about‐genomics/fact‐sheets/DNA‐Sequencing‐Costs‐Data.

[23] B. E. Slatko , A. F. Gardner , and F. M. Ausubel , “Overview of Next‐Generation Sequencing Technologies,” Current Protocols in Molecular Biology 2018, 122 (1), 59, 10.1002/cpmb.59. PMC6020069 29851291

[24] D. Paunescu , M. Puddu , J. O. B. Soellner , P. R. Stoessel , and R. N. Grass , “Reversible DNA Encapsulation In Silica to Produce ROS‐Resistant and Heat‐Resistant Synthetic DNA Fossils',” Nature Protocols 2013, 8 (12), 2440–2448, 10.1038/nprot.2013.154. 24202557

[25] “Deutscher Bundestag,” accessed February 24, 2025, https://www.bundestag.de/gg/grundrechte.

[26] “DNA of Democracy,” accessed February 24, 2025, https://dna‐of‐democracy.de/#das‐project.

[27] A. Kreye , “Wer die Gedruckte SZ‐Wochenendausgabe Ergreift, Hat das Grundgesetz in den Händen,” Süddeutschede, accessed February 24, 2025, https://www.sueddeutsche.de/politik/grundgesetz‐sueddeutsche‐zeitung‐dna‐erbgut‐1.7252524.

[28] L. C. Meiser , P. L. Antkowiak , J. Koch , et al., “Reading and Writing Digital Data in DNA,” Nature Protocols 2020, 15 (1), 86–101, 10.1038/s41596-019-0244-5. 31784718

[29] H. Kipphan , Handbook of Print Media: Technologies and Production Methods (Springer Science & Business Media, 2001), 10.1007/978-3-540-29900-4.

[30] “Süddeutsche Zeitung Konzeptausgabe—REPUBLIC,” accessed February 24, 2025, https://www.republic.de/newsroom/sueddeutsche‐zeitung‐konzeptausgabe.

[31] K. Johansson , P. Lundberg , and R. Ryberg , A Guide to Graphic Print Production (Wiley, 2007).

[32] J. M. Gaspar , “NGmerge: Merging Paired‐End Reads via Novel Empirically‐Derived Models of Sequencing Errors,” BMC Bioinformatics 19, no. 1 (2018): 536, 10.1186/s12859-018-2579-2. 30572828 PMC6302405

[33] A. L. Gimpel , W. J. Stark , R. Heckel , and R. N. Grass , “A Digital Twin for DNA Data Storage Based on Comprehensive Quantification of Errors and Biases,” Nature Communications 2023, 14 (1), 6026, 10.1038/s41467-023-41729-1. PMC10533828 37758710

[34] D. A. Shagin , I. A. Shagina , A. R. Zaretsky , et al., “High‐Throughput Assay for Quantitative Measurement of PCR Errors,” Scientific Reports 7, no. 1 (2017): 2718, 10.1038/s41598-017-02727-8. 28578414 PMC5457411

[35] V. Potapov and J. L. Ong , “Examining Sources of Error in PCR by Single‐Molecule Sequencing,” PLoS One 12, no. 1 (2017): 0169774, 10.1371/journal.pone.0169774. PMC5218489 28060945

[36] W. Li and A. Godzik , “Cd‐Hit: A Fast Program for Clustering and Comparing Large Sets of Protein or Nucleotide Sequences,” Bioinformatics 22, no. 13 (2006): 1658–1659, 10.1093/bioinformatics/btl158. 16731699

[37] L. Fu , B. Niu , Z. Zhu , S. Wu , and W. Li , “CD‐HIT: Accelerated for Clustering the next‐Generation Sequencing Data,” Bioinformatics 28, no. 23 (2012): 3150–3152, 10.1093/bioinformatics/bts565. 23060610 PMC3516142

[38] “Best Practices for Low Diversity Sequencing on the NextSeq 500/550 and MiniSeq Systems,” Illumina Knowledge, accessed September 03, 2025 https://knowledge.illumina.com/instrumentation/general/instrumentation‐general‐reference_material‐list/000002882.

[39] S. Filges , P. Mouhanna , and A. Ståhlberg , “Digital Quantification of Chemical Oligonucleotide Synthesis Errors,” Clinical Chemistry 67, no. 10 (2021): 1384–1394, 10.1093/clinchem/hvab136. 34459892

[40] Y.‐J. Chen , C. N. Takahashi , L. Organick , et al., “Quantifying Molecular Bias in DNA Data Storage,” Nature Communications 11, no. 1 (2020): 3264, 10.1038/s41467-020-16958-3. PMC7324401 32601272

[41] V. Gowda , “DNA‐Encoded Music: Gesamtkunstwerk or Gimmick?,” Harvard Journal of Law & Technology, accessed January 30, 2026 https://jolt.law.harvard.edu/digest/dna‐encoded‐music‐gesamtkunstwerk‐or‐gimmick.

[42] B. P. Kuiper , R. C. Prins , and S. Billerbeck , “Oligo Pools as an Affordable Source of Synthetic DNA for Cost‐Effective Library Construction in Protein‐ and Metabolic Pathway Engineering,” Chembiochem 23, no. 7 (2022): 202100507, 10.1002/cbic.202100507. PMC9300125 34817110

[43] P. L. Antkowiak , J. Lietard , M. Z. Darestani , et al., “Low Cost DNA Data Storage Using Photolithographic Synthesis and Advanced Information Reconstruction and Error Correction,” Nature Communications 11, no. 1 (2020): 5345, 10.1038/s41467-020-19148-3. PMC7582880 33093494

[44] L. Ceze , J. Nivala , and K. Strauss , “Molecular Digital Data Storage Using DNA,” Nature Reviews Genetics 20, no. 8 (2019): 456–466, 10.1038/s41576-019-0125-3. 31068682

[45] Y. Erlich , “A Vision for Ubiquitous Sequencing,” Genome Research 25, no. 10 (2015): 1411–1416, 10.1101/gr.191692.115. 26430149 PMC4579324

[46] C. T. Clelland , V. Risca , and C. Bancroft , “Hiding Messages in DNA Microdots,” Nature 399, no. 6736 (1999): 533–534, 10.1038/21092. 10376592

[47] S. L. Shipman , J. Nivala , J. D. Macklis , and G. M. Church , “CRISPR–Cas encoding of a digital movie Into the genomes of a population of living bacteria,” Nature 547, no. 7663 (2017): 345–349, 10.1038/nature23017. 28700573 PMC5842791

[48] L. Anavy , I. Vaknin , O. Atar , R. Amit , and Z. Yakhini , “Data Storage in DNA With Fewer Synthesis Cycles Using Composite DNA Letters,” Nature Biotechnology 37, no. 10 (2019): 1229–1236, 10.1038/s41587-019-0240-x. 31501560

