# Supplementary material for: Large‐Scale Distribution of Physical Data Using DNA‐of‐Things Technology in Newspaper Printing
Source: Small. 2026 Mar 31;22(29):e11931. doi: 10.1002/smll.202511931 (PMC13206244; doi:10.1002/smll.202511931)
Supplement: Supplementary file 1 — Supporting File: smll73270‐sup‐0001‐SuppMat.docx. [file SMLL-22-e11931-s001.docx]

**Supporting Information**

Large-Scale Distribution of Physical Data Using DNA-of-Things Technology in Newspaper Printing

Francesca Granito, Andreas L Gimpel, Wendelin J Stark, Reinhard Heckel and Robert N Grass*

# **Supporting Note S1**

To assess the role of inhibition during extraction, samples of different printed sizes (two words, two letters, and two dots) were analyzed with and without the addition of blank newspaper paper. Blank paper from the same page was added to selected samples to reach a total mass of 40 mg, matching the mass used for text and figure samples. qPCR analysis showed consistently lower apparent DNA concentrations in samples supplemented with blank paper, indicating matrix-derived inhibition likely originating from paper pre-treatment additives (Supporting Figure S1-2). The comparative analysis of the original and improved extraction processes, with and without purification steps, is shown in Supporting Figure S3.

# **Supporting Note S2**

Error patterns and biases in the sequencing data were analyzed using a clustered analysis pipeline that takes into account both forward and reverse reads, after merging and clustering(Supporting Figure S7). This approach reflects the errors encountered by the codec during the complete decoding process. Importantly, the results represent only the errors that persist in the consensus sequences after clustering, and not the raw read-level errors in the sequencing data. The error analysis of sequencing data, was performed with DDS-Pipeline (github.com/fml-ethz/dds-pipeline).Read clustering by CD-HIT^[1,2]^, and multiple-sequence alignment by kalign^[3]^. The error analysis used the Python package dt4dds^[4]^

# **Supporting Note S3**

The long-term stability of DNA encapsulated in silica has been demonstrated in previous work^[5]^, where silica encapsulation was shown to slow DNA degradation under harsh environmental conditions. In the study, accelerated thermal ageing experiments were used to extrapolate preservation timescales extending to centuries under ambient storage conditions, highlighting the effectiveness of the silica encapsulation as a chemical and physical barrier against hydrolysis and oxidative damage^[5]^. In line with these findings, the stability of encapsulated DNA within printed paper was evaluated using accelerated ageing protocols designed to simulate long-term environmental stress. Thermal ageing experiments were conducted for six days at 70 °C under controlled relative humidity (50%), confirming that silica encapsulation effectively protects DNA from degradation over extended timescales, including after deposition within printed paper, when compared to non-encapsulated DNA controls. For the thermal ageing study, two categories of samples were analyzed at each time point: (i) non-encapsulated DNA encoding Grundgesetz (20 ng of dried DNA), (ii) 50 mg of printed paper containing DNA-embedded ink printed using an Epson ET-2860 inkjet printer (Supporting Figure S8). In addition to thermal stress testing, UV ageing experiments were performed to evaluate resistance to photochemical degradation, which is particularly relevant for applications involving environmental exposure. For these experiments, samples consisting of 5 µL of non-encapsulated DNA solution encoding Grundgesetz (0.0005 ng µL⁻¹), and 50 mg of printed paper per time point, which were exposed to UVC radiation at 25 °C for 1 h using an 8 W UVC source (Xanitalia PRO 375.740 UV STERIL BLU) operating at 50/60 Hz. Upon UVC irradiation, non-encapsulated DNA exhibited pronounced degradation, consistent with known UVC-induced strand break mechanisms^[6]^. In contrast, encapsulated DNA demonstrated markedly enhanced resistance to UVC exposure. This protective effect is attributed to the combined shielding provided by the silica encapsulation and the presence of ink, which likely absorb and scatter incident UV radiation, thereby reducing the effective UV dose reaching the DNA (Supporting Figure S10). To assess the influence of paper porosity and surface coating, conventional inkjet printing paper was used. Abrasion tests were performed using P120 sandpaper to demonstrate the penetration and retention of silica nanoparticles within the paper fiber matrix. For DNA recovery, 50 mg of printed paper was used for extraction, which was carried out according to the described methods. Results are shown in the Supporting Figure S10.

**Supporting Figures**


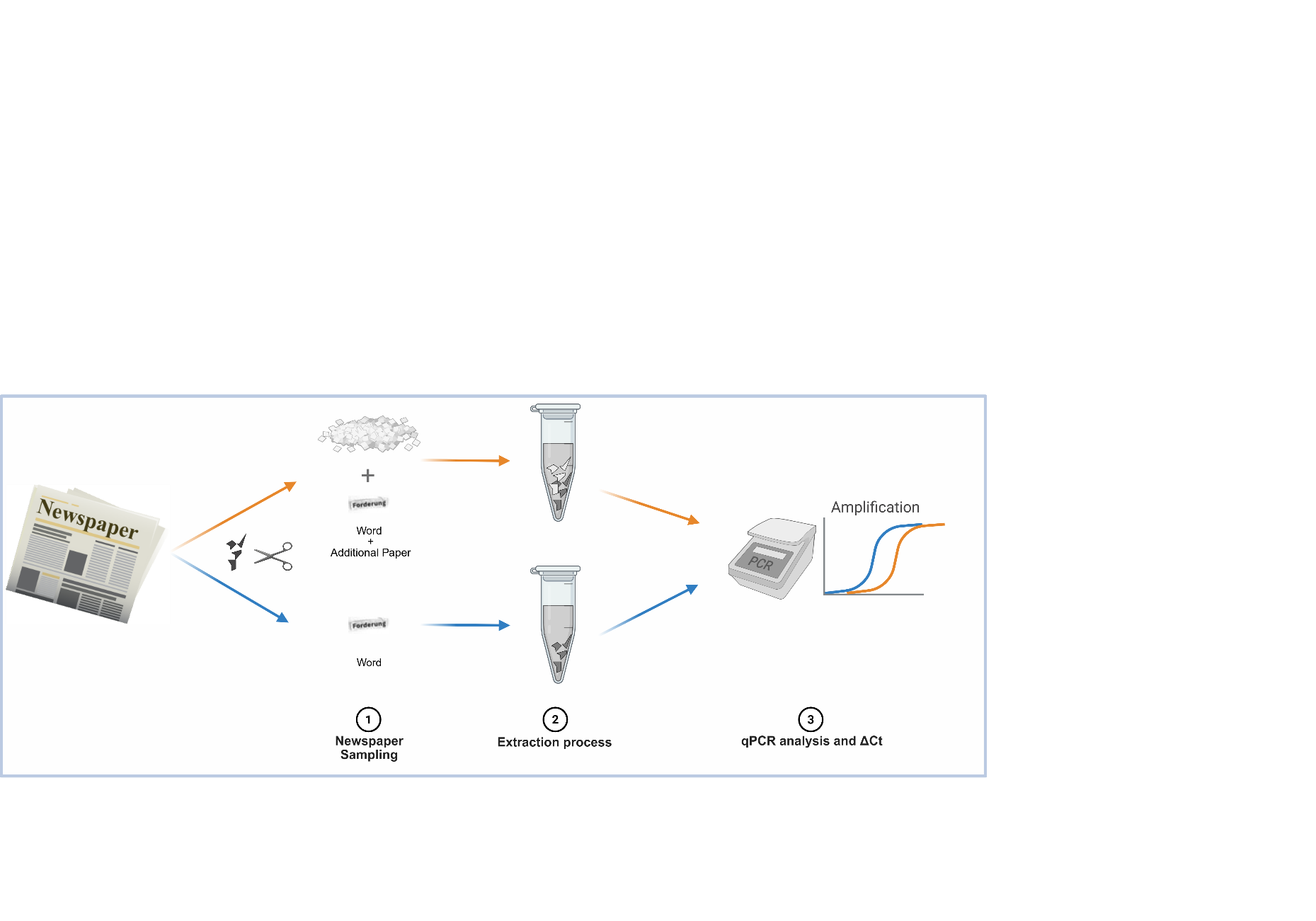


**Supporting Figure S1:** Graphical overview of the inhibition process. Two samples of identical length were used to perform this experiment e.g. two words. In the extraction process a) additional paper selected from the same page was added. In b) the extraction process was performed without any additional paper. Consequently, the extraction process was carried out as illustrated in the methods, and qPCR analysis was performed. Partially created in BioRender.


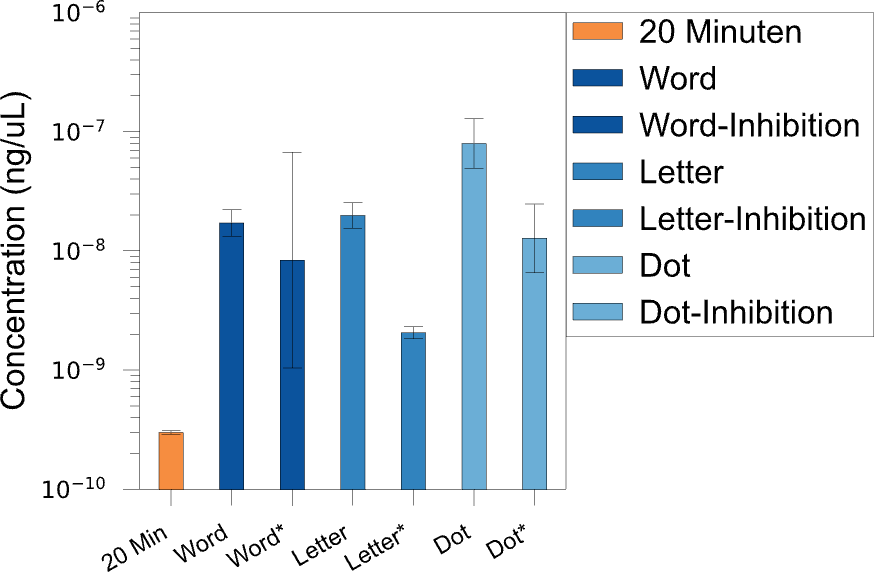


**Supporting Figure S2:** Comparison of the concentration (ng/µl) of samples with/without inhibition generated by the presence of additional paper. Three sets of samples were analysed respectively, two words, two letters, and two dots. The newspaper “20minuten” was used as a negative control.


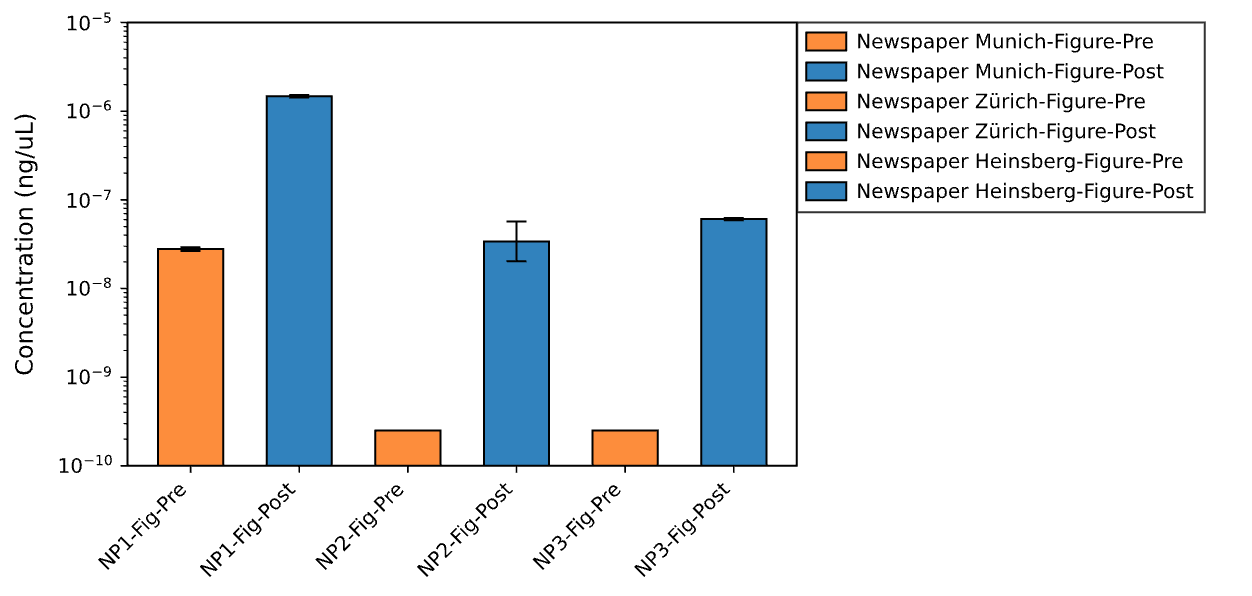


**Supporting Figure S3:** Comparative Analysis of Extraction Processes: Original vs. Improved. Different extraction procedure was attempted to recover the DNA oligo pool from the printed material, the quicker, and more straightforward approach for pigment separation and ink extraction omitted any washing or purification steps This included swelling of the paper with a polar solvent to grant nanoparticles access, followed by the separation of the particle suspension from the fibrous paper matrix. However, this approach was unsuccessful. The comparison emphasizing the gains made following the addition of these stages.


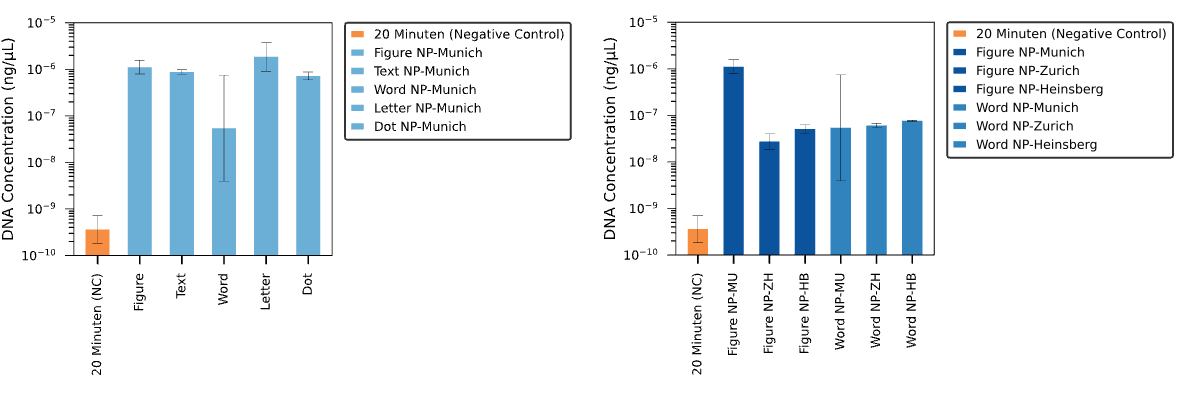


**Supporting Figure S4:** **a)** Concentrations [ng/μl] of DNA oligo pool present in each sample, respectively a Swiss newspaper “20 Minuten” was used as a negative control, followed by different portions of the newspaper from a piece of a figure to a single dot. **b)** Concentrations [ng/μl] of DNA oligo pool present in different portions of two newspapers purchased in distinct geographical areas. NP1–3 correspond to newspapers purchased in Munich, Zurich, and Heinsberg, respectively.


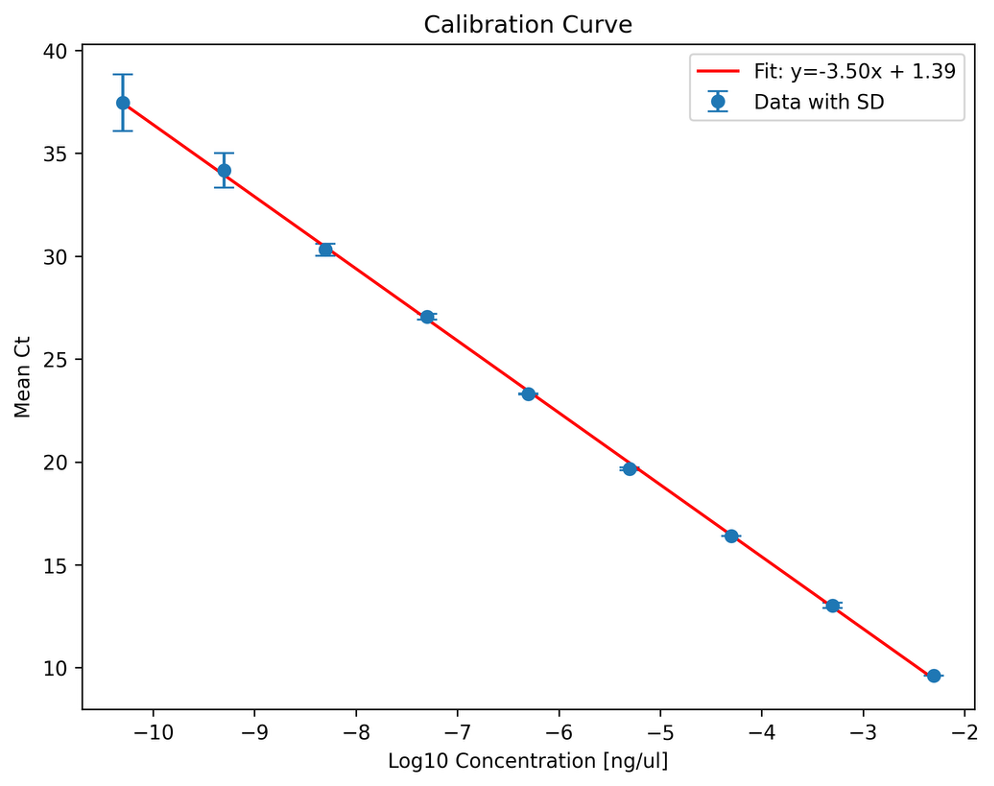


**Supporting Figure S5:** Calibration curve for quantitative PCR: Mean Ct values versus log₁₀-DNA concentrations. Error bars represent the standard deviation of technical replicates. The red line indicates the linear regression fit, with slope and intercept values provided, demonstrating the assay’s linearity and quantitative range.


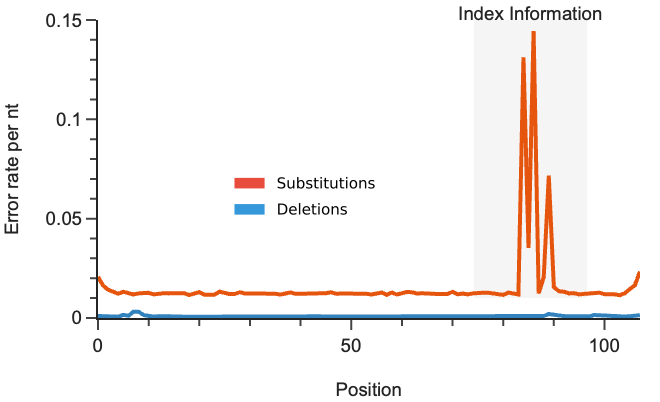


**Supporting Figure S6:** Position-specific error rates averaged over all experiments. The grey box highlights the region containing index information. As the encoder assigns a running number to each sequence,^[7]^ there is lower sequence diversity in this region. This may be ascribed to increased local error rates from the sequencer, which is known to perform less accurately in regions of low diversity.

.
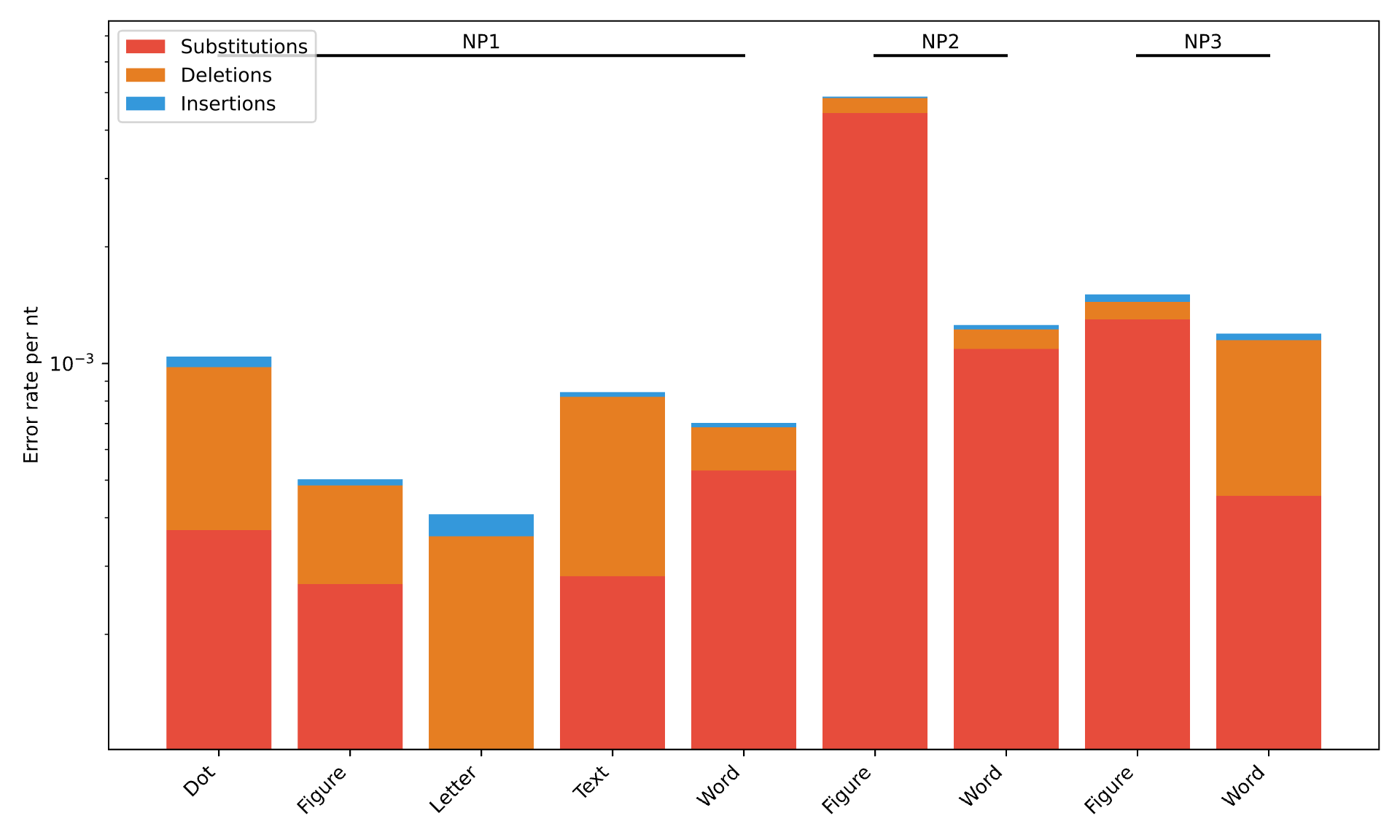


**Supporting Figure S7:** Clustered analysis pipeline. The figure reports the overall error rates for all sub-experiments, presented line by line. For each sub-experiment, the values are given in the following order: match rate, deletion rate, insertion rate, and substitution rate.


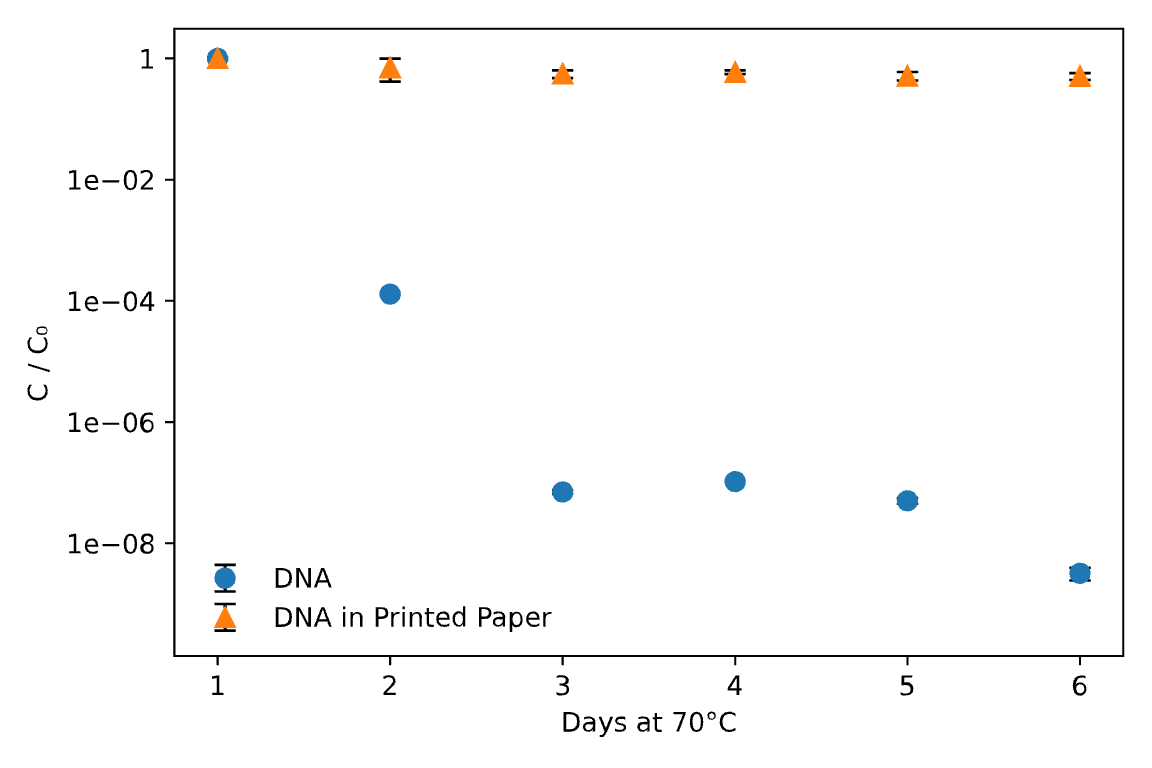


**Supporting Figure S8**: Thermal ageing stability of DNA-embedded ink in printed paper. Samples were subjected to accelerated thermal ageing for six days at 70 °C and 50% relative humidity. Two sample types were analyzed per time point: non-encapsulated DNA encoding Grundgesetz (20 ng dried DNA), and 50 mg of printed paper containing DNA-embedded ink printed using an Epson ET-2860 inkjet printer.


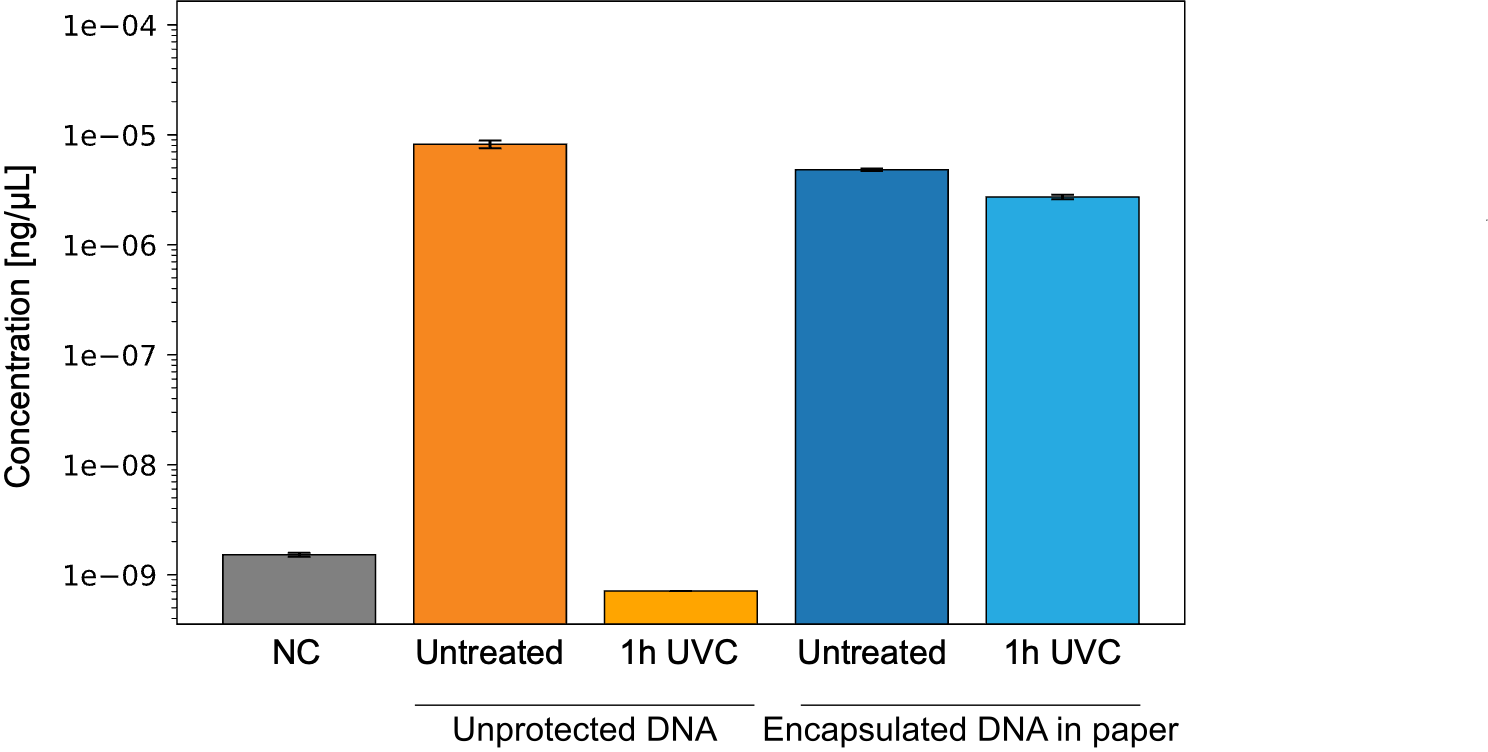


**Supporting Figure S9:** UVC ageing of DNA-embedded ink in printed paper (UVC,8 W, 50/60 Hz, 25 °C, 1 h). Samples: Unprotected DNA (5 µL, 0.0005 ng µL⁻¹), and 50 mg printed paper (Epson ET-2860).


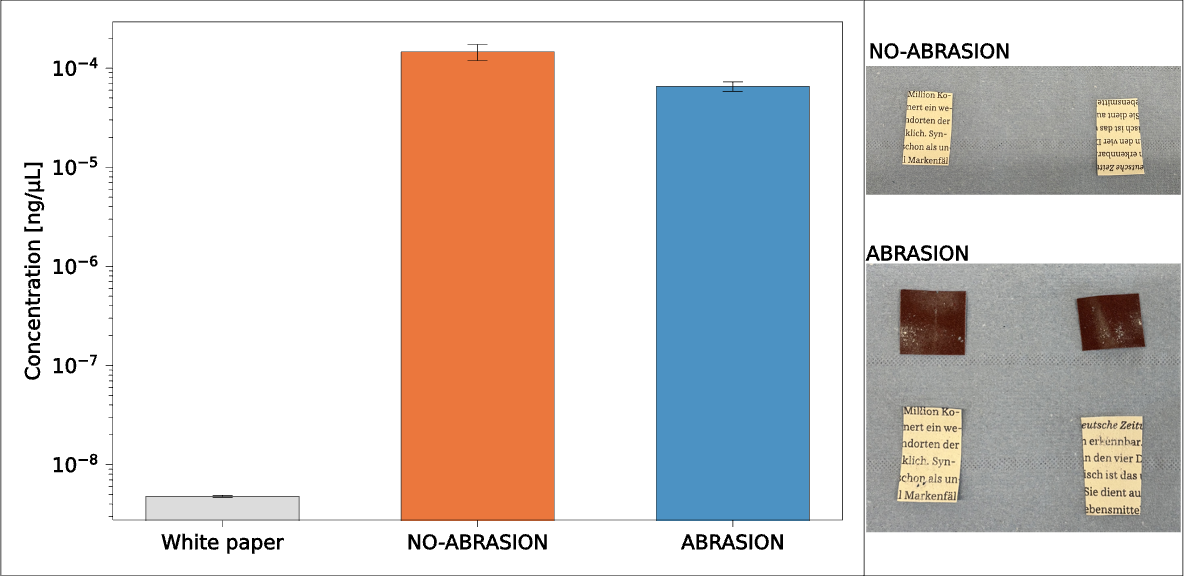


**Supporting Figure S10**: DNA extraction from paper samples of identical mass, with and without abrasion using P120 sandpaper. Unprinted white paper was used as a negative control during the extraction process.


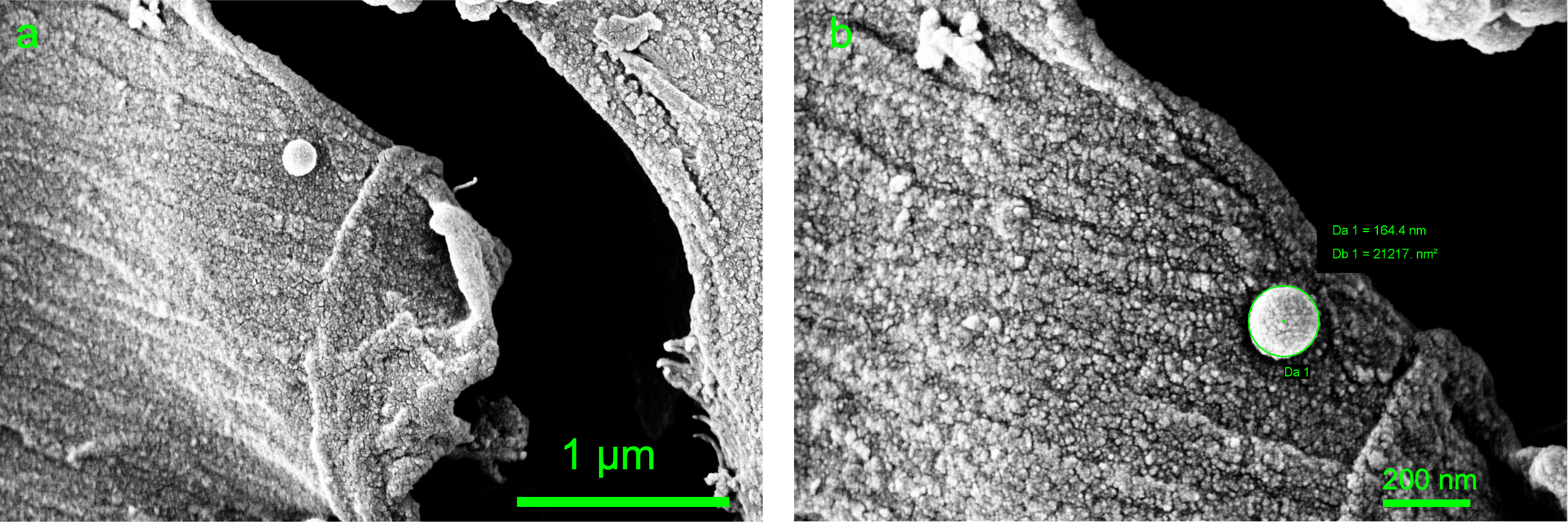


**Supporting Figure S11:** Scanning electron microscope (SEM) images of DNA/SiO₂ particles in ink suspension applied to paper fibers. To evaluate the ink’s behaviour and particle distribution, a portion of blank “20 Minuten”newspaper was tested. The images show the morphology and dispersion of the DNA-loaded silica particles on the paper surface, providing insight into how the ink interacts with the fibers and how the particles are retained after deposition.

# **Supplementary Tables**

**Supporting Table S1:** General Primers.

| Name | Sequence (5’-3’) |
| --- | --- |
| 0F | ACACGACGCTCTTCCGATCT |
| 0R | AGACGTGTGCTCTTCCGATCT |
| 1R | GTGACTGGAGTTCAGACGTGTGCTCTTCCGATCT |
| 1F | ACACTCTTTCCCTACACGACGCTCTTCCGATCT |

**Supporting Table S2:** Primer sequences. Underlined bases indicate Illumina multiplexed index regions.

| Name | Sequence (5’-3’) | INDEX | INDEX-RC |
| --- | --- | --- | --- |
| 2F | AATGATACGGCGACCACCGAGATCTACACTCTTTCCCTACACGACGC | - | - |
| 2RI-GM1 | CAAGCAGAAGACGGCATACGAGATACATCGGTGACTGGAGTTCAGACGTGT | ACATCG | ATCACG |
| 2RI-GM2 | CAAGCAGAAGACGGCATACGAGATCGTGATGTGACTGGAGTTCAGACGTGT | CGTGAT | CGATGT |
| 2RI-GM3 | CAAGCAGAAGACGGCATACGAGATGCCTAAGTGACTGGAGTTCAGACGTGT | GCCTAA | TTAGGC |
| 2RI-GM4 | CAAGCAGAAGACGGCATACGAGATTGGTCAGTGACTGGAGTTCAGACGTGT | TGGTCA | TGACCA |
| 2RI-GM18 | CAAGCAGAAGACGGCATACGAGATGCGGACGTGACTGGAGTTCAGACGTGT | GCGGAC | GTCCGC |
| 2RI-GM19 | CAAGCAGAAGACGGCATACGAGATTTTCACGTGACTGGAGTTCAGACGTGT | TTTCAC | GTGAAA |
| 2RI-GM20 | CAAGCAGAAGACGGCATACGAGATGGCCACGTGACTGGAGTTCAGACGTGT | GGCCAC | GTGGCC |
| 2RI-GM21 | CAAGCAGAAGACGGCATACGAGATCGAAACGTGACTGGAGTTCAGACGTGT | CGAAAC | GTTTCG |
| 2RI-GM22 | CAAGCAGAAGACGGCATACGAGATCGTACGGTGACTGGAGTTCAGACGTGT | CGTACG | CGTACG |
| 2RI-GM23 | CAAGCAGAAGACGGCATACGAGATCCACTCGTGACTGGAGTTCAGACGTGT | CCACTC | GAGTGG |

**Supporting Table S3**: Combination Primers with respective samples.

| Samples | Primers |
| --- | --- |
| 20 Minuten | 2RI-GM1 |
| Newspaper Munich-Figure | 2RI-GM2 |
| Newspaper Munich-Text | 2RI-GM3 |
| Newspaper Munich-Word | 2RI-GM4 |
| Newspaper Munich-Letter | 2RI-GM18 |
| Newspaper Munich-Dot | 2RI-GM19 |
| Newspaper Zürich-Figure | 2RI-GM20 |
| Newspaper Zürich-Word | 2RI-GM21 |
| Newspaper Heinsberg-Figure | 2RI-GM22 |
| Newspaper Heinsberg-Word | 2RI-GM23 |

**Supporting Table S4**: Part per billions (ppb) and Part per millions (ppm) of respectively DNA and silica nanoparticles in printing Ink.

| Part per billions (ppb) of DNA in printing Ink | Concentration  [ng/ul] | Volume  [ul] | DNA [ng] | Ink  [kg] | ppb |
| --- | --- | --- | --- | --- | --- |
|  | 4×10^2^ | 150 | 6×10^4^ | 34 | 1.8 |
| Part per millions (ppm) of nanoparticles in printing Ink | Concentration [mg/ml] | Volume  [ul] | NPs  [mg] | Ink  [kg] | ppm |
|  | 1.4×10^4^ | 150 | 2.1×10^3^ | 34 | 61.8 |

**Supporting Table S5:** Approximate estimation of DNA per ink dot, considering 1 dot of ink 1/10000 of the entire page.

| Approximate quantification of DNA per ink dot | Ink × 1000 Pages  [g] | Dot = 1/10000 Page  [g] | DNA × Dot  [fg] |
| --- | --- | --- | --- |
|  | 81.3 | 8.1×10^-6^ | 14.4 |

**Supporting Table S6:** Comparison of Theoretical and Measured DNA Concentrations per ink dot: This table summarizes the theoretical DNA amount, expected concentration after extraction, measured concentration from PCR, and the calculated DNA amounts in the PCR aliquot and total extract. The comparison shows the measured DNA corresponds to ~77% of the theoretical value, indicating reasonable recovery.

| Theoretical total DNA [fg] | 14 |
| --- | --- |
| Theoretical concentration [ng/µL] | 9.33E-07 |
| Measured concentration [ng/µL] | 7.19E-07 |
| Measured total DNA (15 µL) [fg] | 10.8 |
| Measured vs Theory [%] | 77 |

**References**

[1] W. Li, A. Godzik, “Cd-hit: a fast program for clustering and comparing large sets of protein or nucleotide sequences” *Bioinformatics* **2006**, *22*, 1658–1659.

[2] L. Fu, B. Niu, Z. Zhu, S. Wu, W. Li, “CD-HIT: accelerated for clustering the next-generation sequencing data” *Bioinformatics* **2012**, *28*, 3150–3152.

[3] T. Lassmann, “Kalign 3: multiple sequence alignment of large datasets” *Bioinformatics* **2020**, *36*, 1928–1929.

[4] A. L. Gimpel, A. Remschak, W. J. Stark, R. Heckel, R. N. Grass, **2025**, bioRxiv preprint, DOI: 10.1101/2025.07.11.664297.

[5] R. N. Grass, R. Heckel, M. Puddu, D. Paunescu, W. J. Stark, “Robust Chemical Preservation of Digital Information on DNA in Silica with Error-Correcting Codes” *Angewandte Chemie International Edition* **2015**, *54*, 2552–2555.

[6] E. C. Friedberg, “A brief history of the DNA repair field” *Cell Res* **2008**, *18*, 3–7.

[7] L. C. Meiser, P. L. Antkowiak, J. Koch, W. D. Chen, A. X. Kohll, W. J. Stark, R. Heckel, R. N. Grass, “Reading and writing digital data in DNA” *Nat Protoc* **2020**, *15*, 86–101.
